# *M. leprae* components induce nerve damage by complement activation: identification of lipoarabinomannan as the dominant complement activator

**DOI:** 10.1007/s00401-015-1404-5

**Published:** 2015-03-15

**Authors:** Nawal Bahia El Idrissi, Pranab K. Das, Kees Fluiter, Patricia S. Rosa, Jeroen Vreijling, Dirk Troost, B. Paul Morgan, Frank Baas, Valeria Ramaglia

**Affiliations:** 1 Department of Genome Analysis, Academic Medical Center, 1105 AZ Amsterdam, The Netherlands; 2 Department of Neuropathology, Academic Medical Center, Amsterdam, The Netherlands; 3 Department of Clinical Immunology, Colleges of Medical and Dental Sciences, University of Birmingham, Birmingham, UK; 4 Instituto Lauro de Souza Lima, Bauru, Sao Paulo Brazil; 5 Institute of Infection and Immunity, School of Medicine, Cardiff University, Cardiff, UK

**Keywords:** Leprosy, Complement, Neuropathy, Therapy

## Abstract

Peripheral nerve damage is the hallmark of leprosy pathology but its etiology is unclear. We previously identified the membrane attack complex (MAC) of the complement system as a key determinant of post-traumatic nerve damage and demonstrated that its inhibition is neuroprotective. Here, we determined the contribution of the MAC to nerve damage caused by *Mycobacterium leprae* and its components in mouse. Furthermore, we studied the association between MAC and the key *M. leprae* component lipoarabinomannan (LAM) in nerve biopsies of leprosy patients. Intraneural injections of *M. leprae* sonicate induced MAC deposition and pathological changes in the mouse nerve, whereas MAC inhibition preserved myelin and axons. Complement activation occurred mainly via the lectin pathway and the principal activator was LAM. In leprosy nerves, the extent of LAM and MAC immunoreactivity was robust and significantly higher in multibacillary compared to paucibacillary donors (*p* = 0.01 and *p* = 0.001, respectively), with a highly significant association between LAM and MAC in the diseased samples (*r* = 0.9601, *p* = 0.0001). Further, MAC co-localized with LAM on axons, pointing to a role for this *M. leprae* antigen in complement activation and nerve damage in leprosy. Our findings demonstrate that MAC contributes to nerve damage in a model of *M. leprae*-induced nerve injury and its inhibition is neuroprotective. In addition, our data identified LAM as the key pathogen associated molecule that activates complement and causes nerve damage. Taken together our data imply an important role of complement in nerve damage in leprosy and may inform the development of novel therapeutics for patients.

**Electronic supplementary material:**

The online version of this article (doi:10.1007/s00401-015-1404-5) contains supplementary material, which is available to authorized users.

## Introduction

Leprosy is one of the earliest recorded human infectious diseases. To date, infection with *Mycobacterium leprae* (*M. leprae*) remains the leading cause of infectious neuropathy and disabilities. Despite effective multidrug therapy (MDT), leprosy is still endemic in several parts of the world, especially in Brazil and India. The majority of the infected population remains healthy, whereas a subset of infected individuals develops clinical symptoms, which are associated with host immunity to the bacilli.

The manifestation of the disease displays a broad clinical, histopathological and immunological spectrum, with tuberculoid (TT) and lepromatous (LL) forms at the two poles, and with several intermediate forms including borderline tuberculoid (BT), borderline borderline (BB) and borderline lepromatous (BL) [1]. The BT and TT are paucibacillary (PB), whereas LL, BL, and BB are multibacillary (MB). PB patients show a strong T cell-mediated immunity to *M. leprae*, whereas MB patients show a *M. leprae*-specific cell-mediated response anergy but mount an antibody response, which results in extensive diffuse bacilli-laden skin lesions. In addition to the above-described spectrum of the disease, a percentage of patients, particularly those in the borderline groups during treatment, develop two types of reactions due to changes in their pathogen-specific immune status: type 1 or reversal reaction (RR) and type 2 or erythema nodusum leprosum (ENL). The RR is due to the increased pathogen-specific cell-mediated immunity encountered among BT and BL patients, whereas ENL is seen in BL and LL patients and are thought to be immune complex-mediated [2].

Histologically, skin lesions of paucibacillary patients show T-cell infiltrates and epithelioid giant cells, whereas those of multibacillary patients show a paucity of T-cells and the accumulation of bacilli-laden macrophages. The major pathological hallmark of *M. leprae* infection across the entire disease spectrum is nerve damage.

Nerve damage in leprosy is almost exclusively studied in late disease stages; no published study describes nerve changes at the early stages of the disease. However, epidemiological surveys in endemic areas reported that nerve damage occurs even among the non-diseased leprosy contacts [3], suggesting that nerve damage might commence long before the disease manifests as skin lesions. Indeed, the natural affinity of *M. leprae* for nerve, particularly for Schwann cells, makes it likely that nerve damage starts at a very early stage of infection. However, the mechanisms underlying nerve damage in early disease remain to be elucidated. Understanding the molecular and immunological mechanisms of *M. leprae*-induced nerve damage is a necessary step in the management of leprosy to prevent progression of the infection into an extensive neuropathic condition.

Previous studies in animal models induced by direct interaction of *M. leprae* with nerves have shown that myelin loss and axonal damage can occur in *M. leprae* infection, even in the absence of a functional adaptive immune system [4, 5]. Although the adaptive immune response plays a critical role in the clinical manifestation of the disease, the identification of an adaptive immunity-independent myelin loss suggests the existence of additional mechanisms. We have previously identified an important role of the complement system in myelin loss and axonal injury of the peripheral nerve after acute trauma [6]. The complement system is a key component of the host defense against pathogens but uncontrolled or excessive activation can cause damage to the host. Complement activation can occur via the recognition of antigen–antibody complexes (classical pathway), foreign surfaces (alternative pathway) or bacterial sugars (lectin pathway). Regardless of the trigger, activation results in the cleavage of C3, followed by cleavage of C5 and formation of the membrane attack complex (MAC), which punches holes through the cell membrane resulting in lysis of the target cell. Because activated complement components are soluble and can drift from their site of activation to adjacent areas, MAC can damage adjacent healthy tissue and enhance inflammation [7, 8]. We have shown that formation of the MAC contributes to early clearance of myelin proteins and to axonal damage after traumatic injury of the peripheral nerve [6, 9], while inhibition of MAC formation reduces nerve damage [10] and improves regeneration and functional recovery [11].

Our hypothesis is that complement, specifically the MAC, may play an important role in nerve damage in leprosy. This hypothesis is substantiated by pathological studies, which reported MAC deposits on damaged nerves of LL but not TT leprosy patients [12], pointing to the possibility that complement, and specifically the MAC, plays a role as disease modifier in leprosy. In addition, significant serum complement consumption by *M. leprae* was also reported [13].

In this study, we injected *M. leprae* or its components into the mouse sciatic nerve to induce nerve injury. This model does not recapitulate *M. leprae*-induced neuropathy in man. However, it is a good model to study *M. leprae*-induced loss of axonal components and focal loss of myelin, which we define as nerve damage in this study. Since, we made use of nude mice (NMRI-Foxn1^nu^), which lack functional T and B lymphocytes, we can study the direct role of complement in *M. leprae*-induced nerve damage in the absence of a cellular adaptive immune response. In a first experiment, we demonstrated that *M. leprae* sonicate and its components, particularly lipoarabinomannan (LAM), induce complement activation, which results in MAC deposition, myelin loss and axonal damage of the mouse sciatic nerve. In a second experiment we proved that, in this model, inhibition of MAC formation is neuroprotective. In addition, we explored the extent of complement deposition, including MAC, in a snap-shot of nerve biopsies from patients with full blown leprosy at either of the two poles of the disease spectrum, showing an association between the amount of MAC deposition and LAM immunoreactivity in nerves of leprosy patients. Altogether, our findings strongly point to an important role of complement in nerve damage in leprosy.

## Materials and methods

### Animals

Outbred nude (NMRI-Foxn1^nu^) mice were purchased from Charles River (United Kingdom). The mice were housed under standard pathogen-free conditions and allowed free access to food and water. Female mice, aged between 8 and 12 weeks, were used in all experiments and allowed to acclimatize for at least 1 week prior to the experimental procedures. All experiments complied with national ethical guidelines for the care of experimental animals.

### Bacterial fractions

The following reagents were obtained through BEI resources, NIAID, NIH: whole *M. leprae* sonicate and its fractions, including cell wall, cell membrane, lipoarabinomannan (LAM) and phenolic glycolipid-1 (PGL-1), as well as *M. tuberculosis* sonicate (see Table S1). *M. leprae* was propagated in armadillos.

Both *M. leprae* and *M. tuberculosis* were made non-viable by gamma irradiation before sonication. Gamma-irradiated and sonicated *M. leprae* is referred to in the text and figures as *M. leprae* or sonicated *M. leprae.* Gamma-irradiated and sonicated *M. tuberculosis* is referred to in the text and figures as *M. tuberculosis* or sonicated *M. tuberculosis.*

### Intraneural injection of *M. leprae* sonicate or fractions

Surgical procedures were performed under deep isoflurane anesthesia (2.5 % vol isoflurane, 1 l/min O_2_, and 1 l/min N_2_O). For analgesia, Buprenorphine (0.1 mg/kg, Temgesic^®^, Schering-Plough, The Netherlands) was administered subcutaneously 30 min prior to the surgery. The sciatic nerve was exposed via an incision in the thigh and injected according to the procedure previously described by Rambukkana et al. [4]. Importantly, this pinprick injection by itself does not induce complement activation, myelin loss or axonal damage. Specifically, a microneedle was used to inject the sciatic nerve with a single dose of a solution containing 1 µg of either sonicated *M. leprae* (*n* = 10) or cell membrane (*n* = 7) or LAM (*n* = 5) in a volume of 5 µl. Intraneural injections with equal volume of either phosphate buffer saline (PBS) (*n* = 10) or sonicated *M. tuberculosis* (*n* = 4) were used as controls. In all experiments, the contralateral nerve of each mouse was injected with PBS as internal control.

In addition, sciatic nerves from nude mice that did not receive intraneurial injection were analyzed as controls for the PBS injections. We found no difference in axonal density between non-injected and PBS-injected nerves (data not shown).

The injection site was marked by Indian ink. The skin was sutured and the mice were allowed to recover. At 3 days post-intraneural injections, mice were deeply anesthetized. Blood and liver biopsies were collected for serum analysis and qPCR analysis, respectively. All mice were then euthanized by intracardial perfusion with PBS followed by formalin. The sciatic nerves were collected and post-fixed in formalin for 1 week at 4 °C before they were processed in paraffin for histology, according to standard procedures.

### Mouse tissue preparation and immunohistochemistry

Paraffin-embedded nerves were sectioned at a thickness of 6 μm for the entire length of the nerve, including the site of injection, and mounted on glass slides. Up to 4000 sections per nerve were cut. Three adjacent sections of every 10 were selected and stained for hematoxylin and eosin (H&E) and scored by two independent investigators (NBEI and VR) for damage and accumulation of immune cells. Immunohistochemistry for PGL-1 and/or LAM was used to locate the site of injection in the nerves. Seventy to 80 sections per sciatic nerve were further analyzed by immunohistochemistry to evaluate the axonal, myelin and Schwann cell damage as well as MAC deposition and the extent of endoneurial accumulation of macrophages.

For the immunohistochemistry, sections were deparaffinated and rehydrated. The endogenous peroxidase activity was blocked with 0.3 % H_2_O_2_ in methanol for 20 min at room temperature. Epitopes were exposed by heat-induced antigen retrieval, in either 10 mM sodium citrate buffer (pH 6.0) or 10 mM Tris 1 mM EDTA buffer (pH 9.0) depending on the primary antibody used (see Table S2). Aspecific binding of antibodies was blocked using 10 % normal goat serum (DAKO, Heverlee, Belgium) in PBS for 30 min at room temperature. Primary antibodies were diluted in normal antibody diluent (Immunologic, Duiven, The Netherlands) and incubated for 1 h at room temperature. Detection was performed by incubating the sections in the secondary Poly-HRP-Goat anti-Mouse/Rabbit/Rat IgG (Brightvision Immunologic, Duiven, The Netherlands) antibody diluted 1:1 in PBS for 30 min at room temperature followed by incubation in 3,3-diaminobenzidine tetrahydrochloride (DAB; Vector Laboratories, Burlingame, CA) as chromogen and counterstaining with hematoxylin for 5 min. Sections stained with secondary antibody alone were included as negative controls with each test. After dehydration, slides were mounted in Pertex (Histolab, Gothenburg, Sweden). Images were captured with a light microscope (BX41TF; Olympus,Center Valley, PA) using the Cell D software (Olympus).

For immunofluorescence, the primary antibodies raised in rabbit (see Table S2) were detected with FITC (green, 488 nm)-conjugated goat anti-rabbit IgG (Sigma-Aldrich, Saint Louis, MI) and the primary antibodies raised in mouse were detected with Cy3 (red, 560 nm)-conjugated goat anti-mouse IgG (Sigma-Aldrich, Saint Louis, MI). Sections were counterstained with 4.6-diamidine-2-phenylindole dihydrochloride (DAPI, Sigma-Aldrich) (blue, 280 nm), air dried and mounted in Vectashield (Vector, Burlingame, CA). Images were captured with a digital camera (DFC500; Leica) on a fluorescence microscope (DM LB2; Leica, Wetzlar, Germany).

### Measurement of human serum complement consumption by *M. leprae*

Blood from healthy volunteers was collected by venepuncture and allowed to clot on ice. The serum was separated by centrifugation at 5000×*g* at 4 °C for 10 min and assayed immediately. 50 µl of serum was incubated with equal volume of either whole *M. leprae* sonicate (1 × 10^9^ cells), referred to in the text and figures as *M. leprae*, or PBS as control, for 1 h at 37 °C. In the subsequent step, residual human complement activity was tested in triplicate by hemolytic assay according to standard procedures [14, 15].

### ELISA for fluid-phase terminal complement complex (TCC)

Enzyme-linked immunosorbent assay (ELISA) for TCC, was performed on Microlon high-affinity binding plates (Greiner bio one, Frickenhausen, Germany) coated with 2.5 µg of either *M. leprae*, cell wall, cell membrane, LAM or PGL-1 in carbonate buffer (pH 9.6) overnight at 4 °C. Nonspecific binding was blocked with 10 % bovine serum albumin (BSA) (pH 7.4) for 1 h at room temperature. After washing with 0.05 % Tween in PBS, the wells were incubated with 10 % fresh normal human serum (NHS) in dilution buffer (4 mM barbital, 145 mM NaCl, 2 mM CaCl_2_, 1 mM MgCl_2_, 0.3 % BSA, 0.02 % Tween20) for 1 h at 37 °C. After washing, TCC was detected by incubation with a mouse anti-human C5b-9neo monoclonal antibody (aE11 clone, DAKO) (1:100 in dilution buffer). The wells were washed and then incubated with the polyclonal goat anti-mouse Ig HRPO-conjugate (DAKO) (1:2000 in dilution buffer) for 1 h at room temperature. Plates were developed using tetramethylbenzidine (TMB) as substrate and the reaction was stopped using 1 M H_2_SO_4_. The absorbance was measured at 450 nm. The signals were corrected for background by subtracting the absorbance of the controls.

### Identification of complement pathways activated by *M. leprae*

Neutralizing anti-C1q antibody (anti-C1q-85, Sanquin, Amsterdam, The Netherlands) (50 µg/ml), which inhibits the classical pathway of complement, or C1 inhibitor (C1inh; Cetor, Sanquin) (1 µg/µl), which blocks activation of both the classical and lectin pathways, were pre-incubated with 10 % fresh human serum in dilution buffer for 15 min at 37 °C. Mannose-binding lectin (MBL) deficient serum (10 % in dilution buffer) was used as control for lectin pathway activation. Fresh serum pre-incubated with either BSA or EDTA was used as controls. All sera were assayed for *M. leprae*-mediated generation of TCC by ELISA as described above. Microlon high-affinity binding plates (Greiner Bio-One) were coated with 2.5 µg of *M. leprae* in carbonate buffer (pH 9.6) overnight at 4 °C. Coating of the wells with either 1 µg mannan (Sigma, M7504) or 1 µg IgG_1,2,3,4_ (Gammaquin 160 g/l, Sanquin) were included as controls. Blocking of nonspecific binding sites, detection of the TCC and development of the enzymatic HRP reaction were performed as described above. The signals were corrected for background by subtracting the absorbance of the controls.

### C6 antisense oligonucleotide synthesis

The C6 locked nucleic acid (LNA) oligonucleotides were synthesized with phosphorothioate backbones and 5-methyl cytosine residues (medC) by Ribotask (Odense, Denmark) on a Mermade 12™, using 2 g NittoPhase™ (BioAutomation, Irving, Texas). All oligonucleotides were HPLC purified. C6 oligonucleotide (C6 LNA): 5′ A A C t t g c t g g g A A T 3′. Mismatch control oligonucleotide (mismatch LNA): 5′ A T C t t c g c g t g a a T A A 3′. LNA is shown in capital letters and DNA in lowercase.

### Treatment with C6 antisense oligonucleotide

C6 antisense is a LNA-DNA based gap-mer RNase H recruiting oligonucleotide that specifically targets the mRNA of C6, resulting in the degradation of the mRNA thereby stopping the production of C6 protein, ultimately preventing MAC formation.

Mice were treated with either 5 mg/kg of C6 antisense LNA oligonucleotide (*n* = 5) (referred to as C6 LNA) or scrambled mismatch antisense LNA oligonucleotide as control (*n* = 5) (referred to as mismatch LNA) administered by subcutaneous injections for 4 consecutive days followed by 2 days of suspended treatment prior to intraneural injection with *M. leprae*. At 3 days post-intraneural injections, blood, liver and sciatic nerves were collected as described above.

### qPCR for C6

RNA from the liver was isolated using Trizol according to the instructions of the manufacturer (Invitrogen). cDNA was generated using oligo-dT primer and SuperScriptII enzyme (Invitrogen). qPCR was performed using Universal probe primers (Roche) and a Lightcycler 480 (Roche). Primers specific for C6 were used (C6-forward 5′-CAGAGAAAAATGAACATTCCCATTA; C6-reverse 5′-TTCTTGTGGGAAGCTTTAATGAC). Amplification of C6 mRNA was quantified using LightCycler software (Roche Diagnostics). Values were normalized to hypoxanthine–guanine phosphoribosyltransferase mRNA (HPRT-forward 5′-GGTCCATTCCTATGACTGTAGATTTT; HPRT-reverse 5′-CAATCAAGACGTTCTTTCCAGTT). All reactions were done in quadruplicate and qPCR conditions were as recommended by the manufacturer (Roche).

### Human nerve biopsies

Sural or ulnar nerve biopsies (*n* = 12) of leprosy patients with multibacillary (MB, including BL and LL; *n* = 7) or paucibacillary (PB, including TT and BT; *n* = 5) leprosy, classified according to the Ridley–Jopling scale [1], as well as five control nerve biopsies from Brazilian donors, were obtained at hospitalization at the Instituto Lauro de Souza Lima, Bauru, Sao Paulo, Brazil according to diagnostic procedures (Table S3). The nerve biopsies were chosen randomly from routine pathology from patients with active disease (duration from 6 to 15 months) and chronically inflamed tissues. The control biopsies were from non-leprosy individuals with an unrelated peripheral nerve complaint requiring microsurgery. These specimens were made available by Dr. Marcos Virmond and were found to be devoid of any evidence of infection. Informed consent for the use of diagnostic tissue for research purposes was obtained from the patients.

Briefly, the nerves were fixed in 10 % formalin immediately after dissection and were processed according to standard procedures for embedding in paraffin. Paraffin section of 6 μm thickness were cut using a microtome and mounted on glass slides for further pathological analysis. The immunohistochemistry on the human nerve biopsies was performed essentially as described above for mouse tissue.

### Quantitative analysis of immunohistochemistry on mouse and human nerves

All quantitative analyses of immunohistochemistry were performed with the Image Pro Plus software version 7 (Media Cybernetics Europe, Marlow, UK) by blinded investigators. Digital images of the immunostainings were captured with a light microscope (BX41TF, Olympus) using the Cell D software (Olympus). Images of 20× magnification, covering the complete nerve biopsy were quantified. The surface area stained is expressed as percentage of total area examined. For the mouse nerves error bars represent the standard deviation and for the human nerves error bars indicate standard error of the mean.

### Statistical analysis

Student’s *t* test was performed for statistical analysis comparing two groups. For comparison of more than two groups One way ANOVA with Bonferroni multiple comparison post hoc test was used. Changes were considered statistically significant for *p* ≤ 0.05. For the correlation analysis we included a selection of paucibacillary and multibacillary nerves for which serial sections stained for LAM, MAC and C3d were available. Shapiro–Wilk normality test was performed before using Pearson’s correlation, to determine whether the data was normally distributed.

## Results

### *M. leprae* sonicate induces complement deposition and nerve damage in vivo

To determine whether *M. leprae* induces complement deposition and nerve damage in vivo, sonicates of *M. leprae* or *M. tuberculosis* as a control mycobacterial species were injected into the sciatic nerves of nude (NMRI-Foxn1^nu^) mice. The use of nude mice, which lack functional T and B lymphocytes, allowed us to study the direct role of complement in *M. leprae*-induced nerve damage in the absence of a cellular adaptive immune response. Nerves were analyzed at 3 days post-injection.

**Fig. 1:**
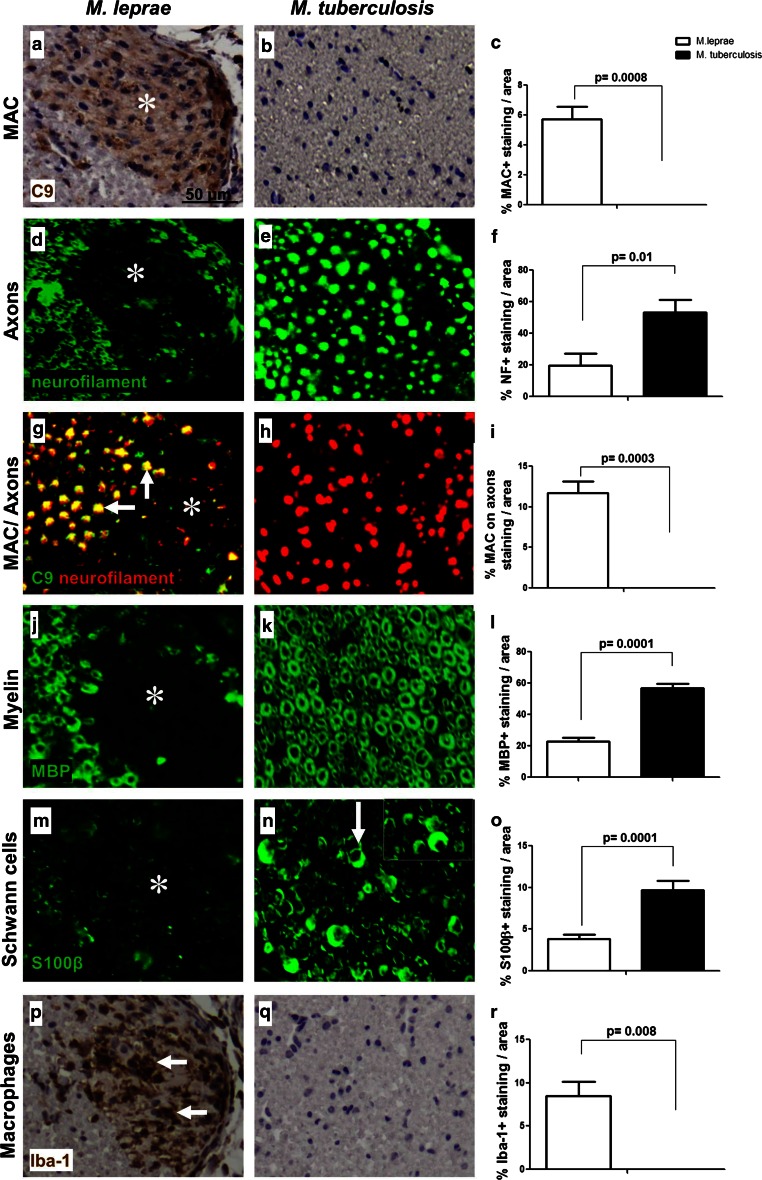
*M. leprae* induces complement deposition and nerve damage in vivo. Immunohistochemistry and quantification for C9 detecting MAC (**a**–**c**), neurofilament detecting axons (**c**–**f**), co-localization of MAC and axons (**g**–**i**), MBP detecting myelin (**j**–**l**), S100β detecting Schwann cells (**m**–**o**), or Iba-1 detecting macrophages (**p**–**r**) in cross sections of mouse sciatic nerves at 3 days post-injection with either *M. leprae* (**a**, **d**, **g**, **j**, **m**, **p**) or *M. tuberculosis* (**b**, **e**, **h**, **k**, **n**, **q**), showing a significant higher amount of MAC immunoreactivity (**a**, *asterisk*) (**c**, Student’s *t* test: *p* = 0.0008), axonal damage (**d**) and loss (**d**, *asterisk*) (**f**, Student’s *t* test: *p* = 0.01), MAC deposited on axons (**g**, *arrows*) (**i**, Student’s *t* test: *p* = 0.0003) and axonal debris (**g**, *asterisk*), myelin loss (**j**, *asterisk*) (**l**, Student’s *t* test: *p* = 0.0001), loss of S100β expression on Schwann cells (**m**, *asterisk*) (**o**, Student’s *t* test: *p* = 0.0001) and accumulation of macrophages (**p**, *arrows*) (**r**, Student’s *t* test: *p* = 0.008) in *M. leprae*-injected nerves compared to *M. tuberculosis*-injected nerves where no MAC deposition and nerve damage was detected (**b**, **e**, **h**, **k**, **n**, **q**). The *arrow* in (**n**) points to the normal moon-shaped appearance of S100β-positive Schwann cells

Intraneural injection of whole *M. leprae* sonicate induced deposition of C9 (a marker for MAC) at the site of injection (Fig. 1a), whereas injection of *M. tuberculosis* sonicate did not (Fig. 1b), *p* = 0.0008 (Fig. 1c). *M. leprae*-induced complement activation was accompanied by axonal damage, as shown by the loss of neurofilament staining in the *M. leprae*-injected (Fig. 1d) but not in the *M. tuberculosis*-injected nerves (Fig. 1e), *p* = 0.01 (Fig. 1f). In the *M. leprae*-injected nerves, C9 deposition was found to localize on neurofilament–positive axons (Fig. 1g, arrows), indicating that MAC attacks the axons in the *M. leprae*-injected nerve but not in the *M. tuberculosis*-injected nerve (Fig. 1h), *p* = 0.0003 (Fig. 1i). The C9 and neurofilament immunoreactivity in the *M. leprae*-injected nerves extended beyond the injection site, some regions around the injection site show reduced neurofilament staining and show no co-localization with C9 deposition (Fig. 1g, asterisk), indicating that macrophages might already have cleared the debris.

The *M. leprae* injection also resulted in loss of immunoreactivity for myelin basic protein (MBP) (Fig. 1j, asterisk), and loss of the S100β Schwann cell marker (Fig. 1m); these changes were not observed in *M. tuberculosis*-injected nerves (Fig. 1k, n), *p* = 0.0001 (Fig. 1l, o). Further, accumulation of macrophages (Iba-1) at the site of injection was observed in *M. leprae*-injected (Fig. 1p) but not in *M. tuberculosis*-injected nerves (Fig. 1q), *p* = 0.008 (Fig. 1r). To additionally control for the possibility that the injection per se may induce nerve damage, the contralateral sciatic nerve of each mouse was injected with PBS. These nerves showed no signs of axonal loss, no loss of the myelin protein MBP, no loss of immunoreactivity for the S100β Schwann cell marker and no deposition of C9 (data not shown).

These data show that the changes observed in the *M. leprae*-injected nerves are antigen-specific and are not the result of the injection per se.

### The *M. leprae* component lipoarabinomannan (LAM) is a dominant complement activator and induces nerve damage in vivo

**Fig. 2:**
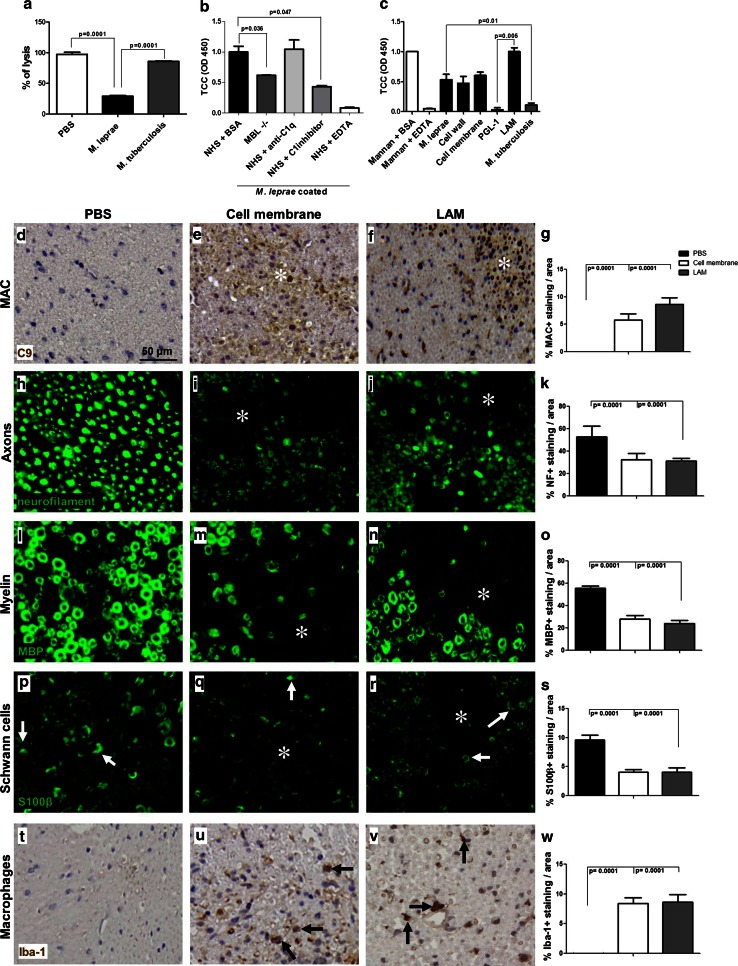
The *M. leprae* component lipoarabinomannan (LAM) is the dominant complement activator and induces nerve damage in vivo. **a** Haemolytic assay of normal human serum (NHS) pre-incubated for 1 h at 37 °C with either *M. leprae* sonicate (5 µg/µl) or *M. tuberculosis* sonicate (5 µg/µl) or PBS as controls, showing significantly decreased haemolytic activity in NHS pre-incubated with *M. leprae* but not with *M. tuberculosis* or PBS, demonstrating complement consumption by *M. leprae*. **b** ELISA for MAC generation on *M. leprae* sonicate (2.5 µg)-coated plates incubated with either mannose binding lectin deficient (MBL^−/−^) serum (to test for the contribution of the lectin pathway) or NHS in the presence of the neutralizing anti-C1q antibody (to test for the contribution of the classical pathway) or C1 inhibitor (to test for the combined contribution of the lectin and classical pathways) or BSA as control, showing a significant reduction of MAC formation in the MBL^−/−^ serum and NHS supplemented with the C1 inhibitor, but not by the neutralizing anti-C1q antibody, demonstrating complement activation by *M. leprae* via the lectin pathway. **c** ELISA for TCC generation in NHS on plates coated with either *M. leprae* sonicate (2.5 µg) or its cellular fractions, including cell membrane (2.5 µg), the inner cell wall component lipoarabinomannan (LAM) (2.5 µg) or the outer cell wall component phenolic glycolipid 1 (PGL-1) (2.5 µg), showing that all components except PGL-1 result in TCC generation. **d**–**r** Intraneural injections of cell membrane or LAM induce complement deposition and nerve damage in vivo. Immunohistochemistry and quantification for C9 detecting MAC (**d**–**g**), neurofilament detecting axons (**h**–**k**), MBP detecting myelin (**l**–**o**), S100β detecting Schwann cells (**p**–**s**) or Iba-1 detecting macrophages (**t**–**w**) in cross sections of mouse sciatic nerves at 72 h post-injection with either PBS (**d**, **h**, **l**, **p**, **t**), cell membrane (**e**, **i**, **m**, **q**, **u**) or LAM (**f**, **j**, **n**, **r**, **v**), showing a significant higher amount of MAC deposition (**e**, **f**, *asterisks*) (**g**, One way ANOVA test: *p* = 0.0001; *p* = 0.0001), axonal damage (**i**, **j**, *asterisks*) (**k**, One way ANOVA test: *p* = 0.0001; *p* = 0.0001), loss of myelin proteins (**m**, **n**, *asterisks*) (**o**, One way ANOVA test: *p* = 0.0001; *p* = 0.0001), loss of S100β expression on Schwann cells (**q**, **r**, *asterisks*) (**s**, One way ANOVA test: *p* = 0.0001; *p* = 0.0001) and accumulation of macrophages (**u**, **v**, *arrows*) in cell membrane- and LAM-injected nerves compared to PBS-injected nerves where no signs of MAC deposition (**d**), undamaged nerve morphology (**h**, **l**), preserved S100β expression (**p**) and a paucity of endoneurial macrophages (**t**) were observed

To determine whether *M. leprae* sonicate is a direct activator of human complement, we tested the capacity of *M. leprae* to induce complement consumption in normal human serum (NHS). Complement consumption was measured in an antibody-induced complement-mediated erythrocyte lysis assay [14]. Pre-incubation of NHS with *M. leprae* significantly reduced haemolysis in this assay compared to PBS pre-incubated controls (67 % reduction; *p* = 0.0001), suggesting that complement was consumed by *M. leprae* (Fig. 2a). Pre-incubation of NHS with *M. tuberculosis* did not significantly reduce haemolysis compared to PBS, indicating that *M. tuberculosis* sonicate, unlike *M. leprae,* is not a strong activator of complement (Fig. 2a).

To confirm that reduction of haemolysis was the result of complement consumption by *M. leprae* rather than inhibition of complement activation, we performed ELISA to detect formation of the MAC in its soluble form, the terminal complement complex (TCC), in human serum added to plates coated with *M. leprae.* In the same experiment, we aimed to identify which complement pathway(s) are activated by *M. leprae* by pre-incubating NHS with either the anti-C1q neutralizing antibody to block the classical pathway or the C1 esterase-inhibitor (C1inh; Cetor), to block both the classical and the lectin pathways. MBL-deficient (MBL^−/−^) serum was also used to test for complement activation via the MBL-dependent lectin pathway. Both, the MBL^−/−^ serum and the C1inh-treated NHS on *M. leprae* showed a significant reduction in TCC formation compared to NHS alone (respectively 57 and 65 %, *p* = 0.036 and *p* = 0.047); the anti-C1q antibody had no effect, suggesting that *M. leprae* activates complement via the lectin pathway (Fig. 2b). As controls, we measured activation of the classical and lectin pathways on mannan- or IgG_1,2,3,4_-coated plates, respectively. Mannan driven TCC formation was significantly reduced in MBL^−/−^ serum compared to MBL^+/+^ serum control (54 % compared to the control, *p* = 0.0001) (Fig. S1a), while pre-incubation of NHS with the anti-C1q antibody showed significant inhibition of IgG-triggered classical pathway activation and TCC formation (81 % compared to the control, *p* = 0.0038, Fig. S1b).

To identify which components of *M. leprae* are responsible for activation of complement, we performed TCC ELISA in NHS on plates coated with either whole *M. leprae* sonicate, cell wall, cell membrane, PGL-1 or LAM. *M. tuberculosis* sonicate or mannan were used as controls. We found that, except for PGL-1, all *M. leprae* components examined induced TCC formation (Fig. 2c). LAM was a strong inducer, resulting in TCC levels close to mannan control values (*p* = 0.05). In line with the in vivo data (Fig. 1a, b), also in this assay, *M.* *tuberculosis* did not induce TCC formation (*p* = 0.01, Fig. 2c). These in vitro data confirmed the in vivo observations that *M. leprae* specifically activates the complement cascade. In addition, we found that LAM is a dominant complement activator in vitro.

To determine whether the *M. leprae* fractions, that induced TCC formation in vitro also caused MAC deposition and nerve damage in vivo, we injected the cell membrane and the LAM fraction in the sciatic nerve of the nude mice and analyzed and quantified the pathological changes at 3 days post-injection (Fig. 2d–w). Intraneural injection of PBS was used as control. PBS caused no pathological changes in the nerves (Fig. 2d, h, l, p, t). Intraneural injections of cell membrane or LAM caused MAC deposition (Fig. 2e, f), axonal damage (Fig. 2i, j), loss of MBP reactivity (Fig. 2m, n), loss of the Schwann cell marker S100β (Fig. 2q, r) and accumulation of Iba-1 positive macrophages (Fig. 2u, v). Quantification of staining on cell membrane- and LAM injected nerves showed a significantly higher amount of MAC deposition (*p* = 0.0001; *p* = 0.0001, respectively), axonal damage (*p* = 0.0001; *p* = 0.0001, respectively), myelin loss (*p* = 0.0001; *p* = 0.0001, respectively), loss of S100β expression (*p* = 0.0001; *p* = 0.0001, respectively) and accumulation of macrophages (*p* = 0.0001; *p* = 0.0001, respectively) compared to PBS-injected nerves. These findings prove that the *M. leprae* cell membrane and purified LAM cause MAC deposition and nerve damage in vivo.

### MAC inhibition protects against *M. leprae*-induced nerve damage

**Fig. 3:**
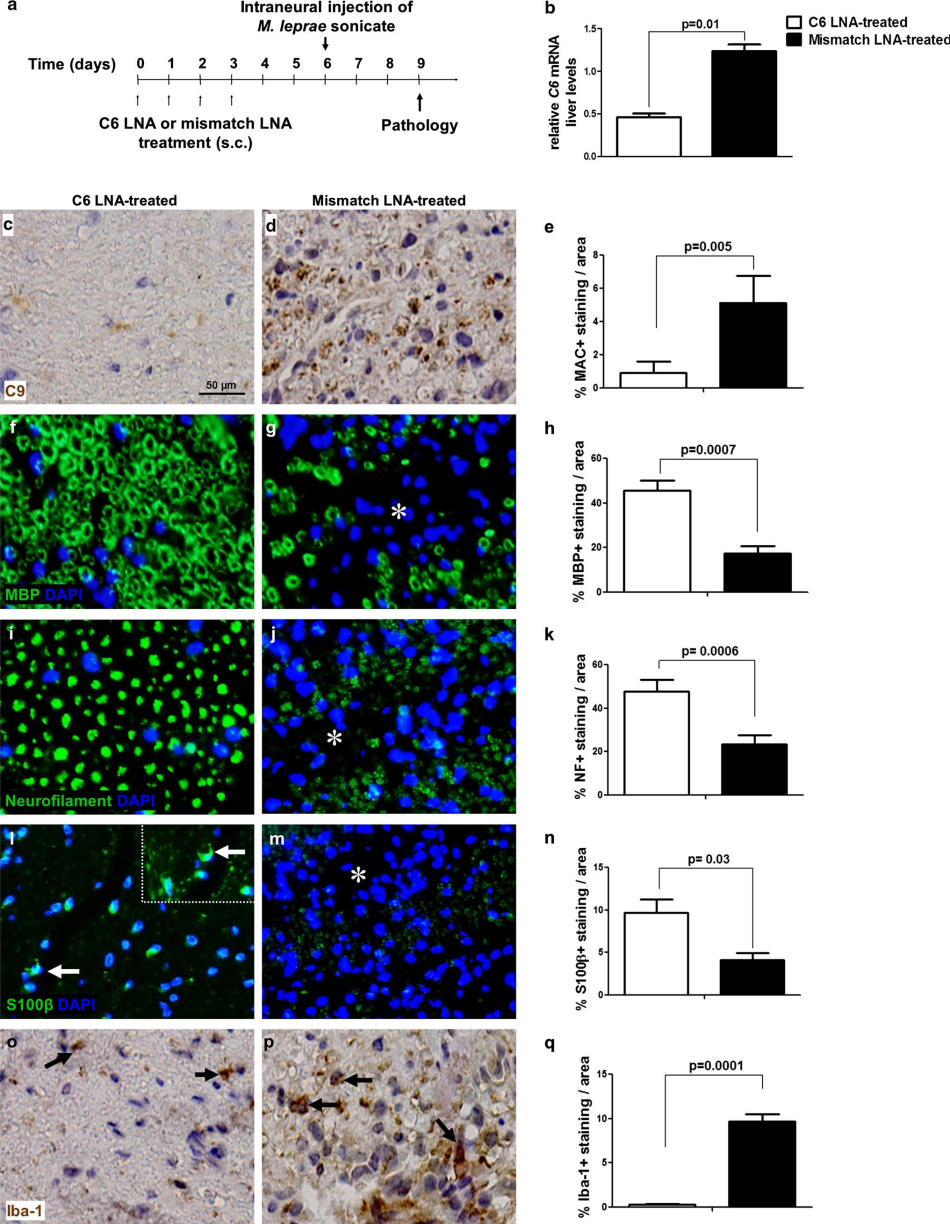
MAC inhibition by C6 antisense therapy protects against *M. leprae*-induced nerve damage. **a** Schedule of treatment and experimental timeline for the C6 antisense therapy. Mice were treated for 4 days with either the C6 LNA (*n* = 5) or the control mismatch LNA (*n* = 5). At day 6, *M. leprae* sonicate was injected into the mouse sciatic nerve. At day 9 (3 days post-injection) mice were sacrificed for determination of C6 mRNA liver levels and pathological analysis. **b** qPCR of liver *C6* mRNA, showing significant lower levels in mice treated with the C6 LNA compared to mismatch LNA-treated controls. Immunohistochemistry and quantification of C9 detecting MAC (**c**–**e**), MBP detecting myelin (**f**–**h**), neurofilament detecting axons (**i**–**k**), S100β detecting Schwann cells (**l**–**n**) or Iba-1 detecting macrophages (**o**–**q**) in cross sections of sciatic nerves from C6 LNA-treated (**c**, **f**, **i**, **l**, **o**) or mismatch LNA-treated (**d**, **g**, **j**, **m**, **p**) mice at 72 h post-injection with *M. leprae* sonicate, showing a significant and robust reduction in MAC deposition (Student’s *t* test: *p* = 0.005) (**e**), intact myelin (Student’s *t* test: *p* = 0.0007) (**h**) and axonal morphology (**k**), S100β expression by Schwann cells (**l**, *arrows*) and reduced accumulation of macrophages (Student’s *t* test: *p* = 0.0001) (**k**) in C6 LNA-treated mice compared to mismatch-treated controls (*asterisks* in **g**, **j**, **m** indicate damaged areas of the mismatch-treated nerves, *arrows* in **p** indicate iba-1 positive macrophages in the mismatch-treated nerves)

To determine the contribution of MAC formation to *M. leprae* sonicate-induced nerve damage in vivo, we treated mice with an antisense LNA-DNA oligonucleotide against C6 for 4 days, starting at 1 week prior to the intraneural injection of *M. leprae* sonicate (Fig. 3a). In the absence of C6, MAC cannot be formed. Quantification of *C6* mRNA in the liver of C6 LNA-treated mice showed a significant 60 % reduction compared to mismatch LNA-treated controls (*p* = 0.01) (Fig. 3b). Such reduction in the amount of *C6* mRNA liver levels is sufficient to block MAC formation, as shown by the significant 80 % reduction of MAC deposits in the nerves of C6 LNA-treated mice compared to mismatch LNA-treated controls at 3 days post-injection (*p* = 0.005) (Fig. 3c–e). In addition, C6 LNA treatment conserved the intact annular nerve morphology and preserved staining of the myelin protein MBP, compared to the collapsed myelin structure and significant loss of myelin MBP immunoreactivity seen in the mismatch LNA-treated animals (*p* = 0.0007) (Fig. 3f–h). Axons were protected from damage in the C6 LNA-treated mice but not in the mismatch LNA-treated animals, which showed a significant loss of neurofilament immunoreactivity (*p* = 0.0006) (Fig. 3i–k). The nerves of C6 LNA-treated mice showed also expression of the Schwann cell marker S100β which had normal appearance as half-moon-shaped profiles (Fig. 3l, arrows), whereas in the mismatch LNA-treated nerves this marker was significantly reduced (*p* = 0.03) (Fig. 3l–n). Lastly, C6 LNA treatment significantly reduced accumulation of intraneural Iba-1 positive macrophages compared to controls (*p* = 0.0001) (Fig. 3o–q). These data show that inhibition of C6 synthesis blocks MAC deposition in the *M. leprae*-injected nerves and prevents the loss of myelin and axonal proteins, protects from the loss of a key Schwann cell marker and reduces accumulation of intraneural macrophages.

### Leprosy nerves are LAM positive and show MAC deposition

**Fig. 4:**
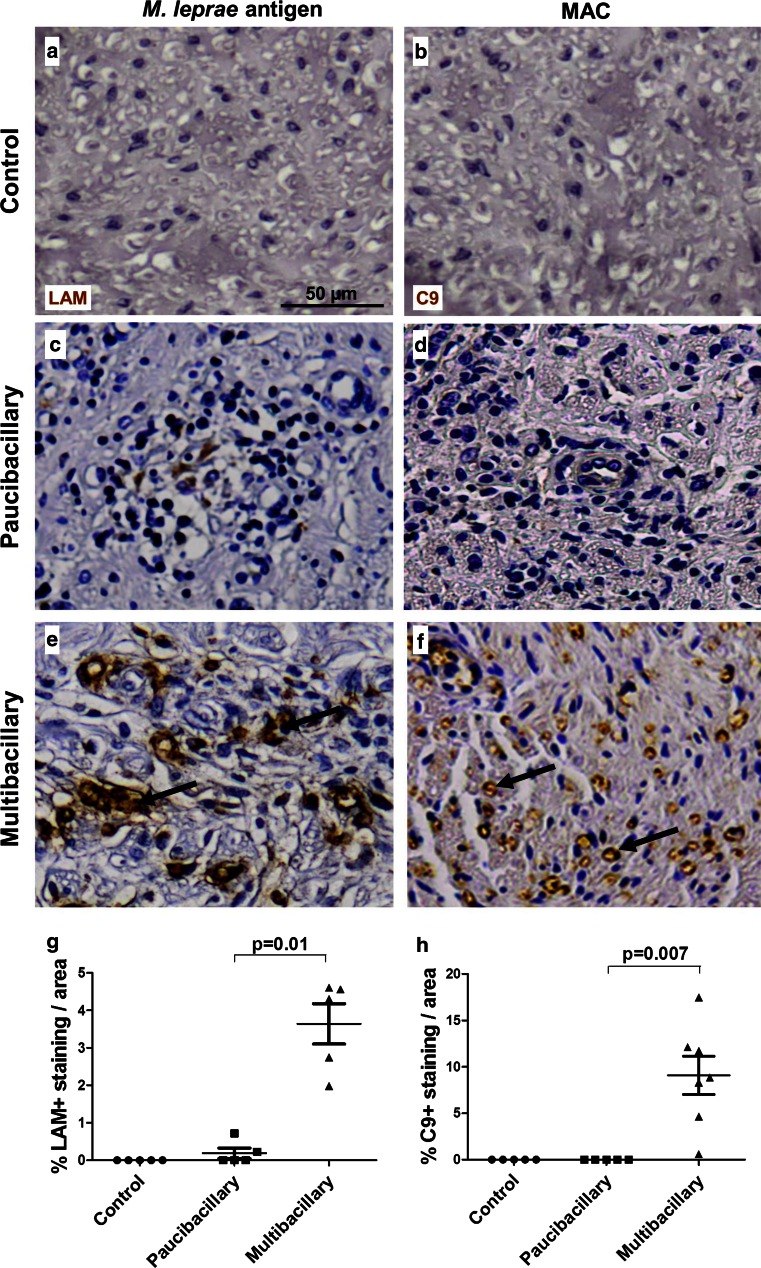
LAM and MAC deposition in nerves of leprosy patients. Immunostaining for the *M. leprae* antigen LAM and C9, detecting MAC, in nerve biopsies of control (**a**, **b**) compared to paucibacillary (**c**, **d**) and multibacillary (**e**, **f**) leprosy patients. The control nerves were negative for LAM (**a**) and MAC (**b**), as expected. Paucibacillary nerves show little immunoreactivity for LAM (**c**) and virtually no MAC deposition (**d**), whereas multibacillary patients show robust staining for LAM (**e**, *arrows*) and MAC (**f**). Quantification of the immunostaining showed that the amount of immunoreactivity for LAM (**g**) and MAC (**h**) is significantly higher in multibacillary compared to paucibacillary nerves (Student’s *t* test paucibacillary vs. multibacillary: LAM, *p* = 0.01; C9, *p* = 0.007). *Error bars* indicate standard error of the mean

To determine the extent of *M. leprae* antigen deposition and to test whether MAC is deposited in the nerve biopsies of leprosy patients, we performed immunohistochemistry for LAM and MAC on nerve biopsies from paucibacillary and multibacillary patients (Fig. 4a–f). These nerves, showed substantial myelin and axonal loss, as demonstrated by quantification of the immunostaining for MBP and SMI31 (Fig. S2). Immunostaining for LAM and MAC were always negative in control nerves (Fig. 4a, b, respectively). Nerves of paucibacillary and multibacillary patients were both positive for LAM (Fig. 4c, e) with the percentage of LAM staining per surface area being significantly higher in multibacillary nerves compared to paucibacillary (*p* = 0.01) (Fig. 4g). Nerves of multibacillary patients also showed substantial MAC deposition (up to 15 % of total area assessed, mean 8 %) (Fig. 4f, h), whereas nerve biopsies from paucibacillary patients were negative for MAC (*p* = 0.007) (Fig. 4d). In line with the robust deposition of MAC, we also found substantial C3d deposition in nerves of multibacillary patients, with the percentage of C3d staining per surface area being significantly higher (>4-fold) than paucibacillary patients (*p* = 0.006) (Fig. S3).

### Complement deposition is associated with *M. leprae* antigen LAM in leprosy lesions

**Fig. 5:**
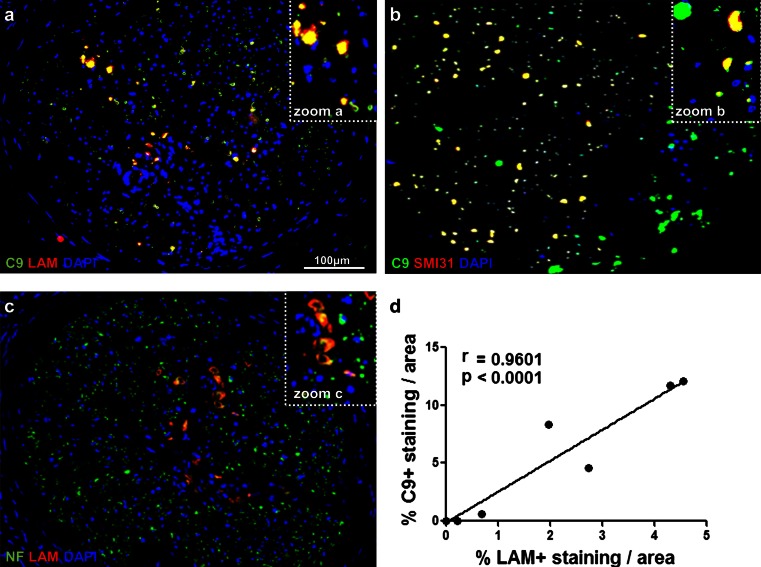
LAM is associated with MAC deposition in nerves of leprosy patients. Immunofluorescent double staining for complement component C9, detecting MAC, and the *M. leprae* antigen LAM, showing co-localization in the nerves of multibacillary patients (**a**). C9 and LAM also co-localized with the SMI31 (**b**) and the neurofilament (NF) (**c**) markers of axons, respectively. The amount of C9 immunoreactivity significantly correlated with the amount of LAM immunoreactivity found in paucibacillary and multibacillary leprosy nerves (Pearson’s correlation, *r* = 0.9601, *p* < 0.0001) (**d**), indicating an association between the extent of *M. leprae* antigen LAM and MAC deposition in leprosy nerves

To determine the location of the deposition of activated complement components in the nerves of multibacillary leprosy patients, we performed immunofluorescent double staining for MAC with the *M. leprae* antigen LAM or markers of axons (SMI31 or pan-neurofilament). We found co-localization of LAM with MAC (Fig. 5a), indicating that complement targets *M. leprae* in the nerve, as expected. Notably, MAC immunoreactivity extended also to LAM-negative nerve areas, which we identified to be axons as shown by the double immunolabeling of C9 with the axonal marker SMI31 (Fig. 5b). In addition, co-localization of LAM with neurofilament (see Table S2), showed that LAM is present in close proximity to axons which show signs of damage, including swelling and degradation (Fig. 5c). Together these data suggest a functional link between complement, the *M. leprae* antigen LAM and axonal changes in leprosy.

To determine whether there is a link between the amount of LAM and the amount of complement activation in the nerves of paucibacillary and multibacillary leprosy patients, we tested whether there is a correlation between the extent of C9 staining and the extent of LAM staining in corresponding nerve areas. We found a highly significant positive correlation between the amount of LAM and MAC (*r* = 0.9601, *p* < 0.0001) in leprosy nerves (Fig. 5d). In line with these findings, we also found a significant correlation between the percentage of complement-immunoreactivity for C3d (*r* = 0.9692, *p* = 0.0003) or C9 (*r* = 0.9682, *p* = 0.0015) and the bacterial index in nerve biopsies of paucibacillary and multibacillary leprosy patients (Fig. S4a, b). Overall these data show a strong link between the presence of *M. leprae* antigen LAM in the nerves and complement activation.

## Discussion

The occurrence of polyneuropathy due to various infectious agents is well recognized in the literature [16]. Among them, nerve damage in leprosy leading to permanent disability still represents an important global health problem. The nerve damage in leprosy is widely regarded as the consequence of adaptive immunity via *M. leprae*-specific T cell activity, persisting long after the patients have completed treatment [17]. However, the nerve damage should be regarded as an early sign of leprosy, because the loss of sensation in patients with suspected leprosy is considered the hall mark of early disease [18]. Despite advances in our knowledge of the pathogenesis of leprosy spectrum, the understanding of the mechanisms of nerve damage and regeneration in leprosy-associated neuropathy remains poor. Progress has been limited by the lack of established experimental models for studying leprosy-induced neuropathy.

Assuming that nerve dysfunction occurs at the onset of effective infection, it can be hypothesized that before the initiation of host adaptive immunity, a direct interaction between the nerve and the infectious agent, could be the initiator of nerve damage which is then compounded by the inflammatory sequel. In support of this hypothesis, literature reports imply that loss of myelin proteins can be induced by *M. leprae* in the absence of lymphocytes in Rag knock out mice [4]. These data suggest the existence of host innate factors that interact with a pathogen-associated molecule (PAM) causing the initial damage. Understanding the molecular mechanisms, which initiate nerve damage in leprosy, is critical for the development of effective therapies aimed at preventing the severe disability in patients.

Response against pathogens, which result in the activation of host’s innate and adaptive factors, is essential for containing the infection, but excessive activation can damage self-tissues. We have previously shown that activation of the complement system, a key component of the host’s immune response, is an important player in the process of nerve damage and regeneration. Specifically, we proved that formation of the membrane attack complex (MAC: comprised of C5b C6 C7 C8 and C9) is essential for rapid Wallerian degeneration of axons in peripheral nerves and inhibition of MAC formation promotes axonal regeneration and recovery of the damaged nerve [6, 9, 11].

In view of these key findings, we undertook the present study in two subsequent steps Firstly, we made use of a mouse model of *M. leprae*-induced nerve injury to elucidated the molecular pathways of the interaction between the nerve and *M. leprae* components. Secondly, we analyzed nerve biopsies of leprosy patients to establish the relevance of our experimental findings in the understanding of the pathology of leprosy neuropathy. The combined data collected from the mouse experiments and from the immunohistopathological analysis of nerve biopsies of leprosy patients, led to our conclusion that lipoarabinomannan (LAM) of *M. leprae* is the dominant PAM, which interacts with the nerve and initiates complement activation resulting in the in situ formation of the MAC, causing nerve damage. We also show that inhibition of MAC formation by antisense oligonucleotide-based therapy protects the nerve from *M. leprae*-induced damage. Therefore we propose that MAC inhibition could form the basis of future development of novel therapeutics for leprosy.

Complement activation in leprosy has been previously associated with immune complexes, pointing to the involvement of the classical pathway of complement in the disease [19]. Our data show that LAM-mediated complement activation is initiated via the lectin pathway, potentially occurring via the binding of MBL or ficolins from the circulation. However, we do not exclude a contribution of other pathways in the pathogenesis of leprosy. We further demonstrated the co-localization of axonal markers with LAM and MAC, which strongly points to the possibility that LAM interacts with an axonal component and activates the complement cascade. Complement activation induced by LAM may trigger a number of events, including activation of neuronal cells, in situ generation of chemokines and chemoattractants, recruitment of inflammatory cells including macrophages, ultimately leading to nerve fragmentation in a similar manner to that seen in Wallerian degeneration [20–22].

The involvement of LAM in the pathogenesis of leprosy-induced neuropathy is also supported by early studies showing that clearance of LAM from granulomas in skin lesions is inefficient. Even after completion of treatment, LAM could still be detected in skin and nerve biopsies from leprosy patients, with clearance of LAM from granulomas in multibacillary lesions being slower than other antigens e.g. PGL-1 [23]. Interestingly, in this work the in situ expression of LAM appeared to be associated with the occurrence of a reactional state. LAM is abundantly present in infiltrating macrophages in lesions of multibacillary patients but not in paucibacillary patients. In the latter case, hardly any macrophage infiltration is seen, instead epitheloid cells are usually present.

LAM, a major pathogen-associated molecule of *M. leprae*, could be the trigger for complement activation and subsequent demyelination. This generates myelin debris, which by itself also activates complement and will attract macrophages [6]. In this way, a vicious cycle occurs. Since LAM can be detected in nerves of leprosy patients accompanied by myelinated axonal loss after multidrug therapy, the signals for myelinated axonal loss might persist even after treatment.

We and others [12] also found that C3d and MAC are abundantly deposited in nerves of multibacillary patients, even in biopsies from patients that have completed treatment.

However, we should emphasize that the analysis of the nerve biopsies of leprosy patients represents a snapshot of the disease pathology at the time when the patient comes into the clinic. Therefore, a temporal course of pathological processes cannot be concluded from the sole analysis of these biopsies. In view of this consideration, the lack of MAC immunoreactivity in the nerves of paucibacillary patients, should be interpreted carefully. Based on the in vitro and in vivo findings reported in this study, we propose that the lack of MAC immunoreactivity in the paucibacillary biopsies is likely due to the fact that in these patients the nerves are severely damaged and MAC-activating debris and LAM are almost completely cleared, resulting in no obvious MAC deposition at the time of biopsy. In line with this interpretation, we also found a strong association between the presence of LAM and MAC deposition in the nerves, which suggests a functional link between these two factors.

Here we investigated the acute effects of the cognate interaction of the nerve with *M. leprae* components which trigger complement activation causing nerve damage. This initial event cannot be studied in humans, because leprosy is a slowly developing chronic inflammatory disease with adaptive immunity in operation, resulting in *M. leprae* disruption, release and subsequent clearance of its components. In such a situation the host may be intermittently exposed to some *M. leprae* components (e.g. LAM) due to inefficient clearance or access of drug into nerves leading to changes in component concentrations, altering the drive to complement activation that may be relevant to the ongoing disease process.

Our model, comprising *M. leprae* intraneurial injections in nude mouse sciatic nerves does not accurately represent human leprosy but rather tests the capacity of *M. leprae* inactivated by gamma irradiation or fractions thereof to activate complement; infection with intact *M. leprae* in the immunocompetent host would not allow such an analysis. Our analysis of human nerve biopsies from leprosy patients allowed us to extrapolate results from the mouse model to man, showing relevance of complement activation to the human disease. Complement was activated in leprosy nerve biopsies, including formation of MAC, capable of damaging myelin and causing lysis of the target cell. Nerve biopsies were available only from established disease so these results only provide a snapshot demonstration of MAC deposition in diseased nerves and do not allow us to conclude that the nerve damage, which occurs early, is mediated by complement activation. A longitudinal study would be important to test the role of MAC in nerve damage early in disease and such a study is currently in progress.

In conclusion, we have shown that LAM is a dominant complement activating *M. leprae* antigen. We also showed, in a model optimized to study the early cognate interaction of *M. lepra*e components with the axon, that this interaction leads to complement activation, myelin loss and axonal damage. Importantly, we proved that inhibition of MAC formation prevented myelin and axonal loss in this model, providing the proof of principle that blocking MAC formation may potentially reduce nerve damage in *M. leprae*-induced neuropathy.

## Electronic supplementary material

Below is the link to the electronic supplementary material.

Supplementary material 1 (PDF 93 kb)

Supplementary material 2 (PDF 280 kb)

Supplementary material 3 (PDF 14 kb)
