# Supplementary material for: M. leprae components induce nerve damage by complement activation: identification of lipoarabinomannan as the dominant complement activator
Source: Acta Neuropathol. 2015 Mar 15;129(5):653–67. doi: 10.1007/s00401-015-1404-5 (PMC4405335; doi:10.1007/s00401-015-1404-5)
Supplement: Supplementary file 1 — Supplementary material 1 (PDF 93 kb) [file 401_2015_1404_MOESM1_ESM.pdf]

***M. leprae* components induce nerve damage by complement activation: Identification of lipoarabinomanan as the dominant complement activator**, Acta Neuropathologica, Nawal Bahia El Idrissi , Pranab K. Das , Kees Fluiter , Patricia S. Rosa, Jeroen Vreijling, Dirk Troost , B. Paul Morgan , Frank Baas and Valeria Ramaglia ; Corresponding author Prof. Frank Baas, email: f.baas@amc.nl.

Table S1. Bacterial fractions (BEI Resources)

**Catalogue number**	**Product Description**
NR-19329	Whole Cell Sonicate of gamma-irradiated -- *Mycobacterium leprae*
NR-19348	Lipoarabinomannan (LAM) -- *Mycobacterium leprae*
NR-19342	Phenolic Glycolipid-1 (PGL-1) -- *Mycobacterium leprae*
NR-19333	Cell Wall Fraction (MLCwA) -- *Mycobacterium leprae*
NR-19331	Cell Membrane Fraction (MLMA) -- *Mycobacterium leprae*
NR-14821	HN878 Gamma-irradiated Whole Cells – *Mycobacterium tuberculosis*

Table S2. Antibody, source, dilution

Antibody	Detects	Source	Dilution
Polyclonal rabbit anti-rat C9 (cross-reacts with human C9)	MAC	Made in house (B.P. Morgan)	1:200’
Polyclonal rabbit anti-human MBP	Myelin	Dako (A0623)	1:100*
Polyclonal rabbit anti-mouse Neurofilament	Axons	Abcam (ab8135)	1:1000*
Polyclonal rabbit anti-mouse S100β	Schwann cells	Dako (Z0311)	1:400*
Polyclonal rabbit anti-mouse Iba-1	Macrophages	Wako (019-19741)	1:200*
Monoclonal mouse anti-*M. leprae* PGL-1	Phenolic glycolipid-1	Made in house (P.K. Das)	1:200’
Polyclonal rabbit anti-human C3d	C3dg	Dako (A0063)	1:200*
Monoclonal mouse anti-LAM	LAM	Made in house (P.K. Das)	1:200’
Monoclonal mouse anti-human phosphorylated neurofilament (clone SMI31)	Axons	Sternberger Monoclonals Inc.	1:1000’

*Antigen retrieval was performed with either 10mM Tris 1mM EDTA pH 9’ or 10mM Sodium Citrate pH 6**

Table S3. Characterization of nerve biopsies and clinical data of leprosy patients and controls

Case	Nerve biopsy	Leprosy type	Gender	Age diagnosis (in years)	Treatment
1	ulnar	-	F	Unknown	-
2	sural	-	F	50	-
3	sural	-	M	54	-
4	sural	-	M	45	-
5	sural	-	F	60	-
6	sural	Paucibacillary	M	65	MDT
7	sural	Paucibacillary	M	Unknown	Unknown
8	ulnar	Paucibacillary	M	59	MDT
9	ulnar	Paucibacillary	M	52	ROM
10	sural	Paucibacillary	F	43	MDT
11	sural	Multibacillary	F	28	DDS
12	sural	Multibacillary	F	43	MDT
13	fibular	Multibacillary	M	Unknown	Unknown
14	ulnar	Multibacillary	M	36	MDT
15	ulnar	Multibacillary	M	27	Unknown
16	sural	Multibacillary	M	43	DDS, MDT
17	ulnar	Multibacillary	M	49	MDT,<br>Thalimidoglutarimide<br>Prednisolone

F, female; M, male; MDT, multidrug therapy; DDS, diamino diphenyl sulphone; ROM, rifampicin, ofloxacin and minocycline.
