# Supplementary material for: M. leprae components induce nerve damage by complement activation: identification of lipoarabinomannan as the dominant complement activator
Source: Acta Neuropathol. 2015 Mar 15;129(5):653–67. doi: 10.1007/s00401-015-1404-5 (PMC4405335; doi:10.1007/s00401-015-1404-5)
Supplement: Supplementary file 2 — Supplementary material 2 (PDF 280 kb) [file 401_2015_1404_MOESM2_ESM.pdf]

***M. leprae* components induce nerve damage by complement activation: Identification of lipoarabinomanan as the dominant complement activator**, Acta Neuropathologica, Nawal Bahia El Idrissi , Pranab K. Das , Kees Fluiter , Patricia S. Rosa, Jeroen Vreijling, Dirk Troost , B. Paul Morgan , Frank Baas and Valeria Ramaglia ; Corresponding author Prof. Frank Baas, email: f.baas@amc.nl.

**Fig S1.**

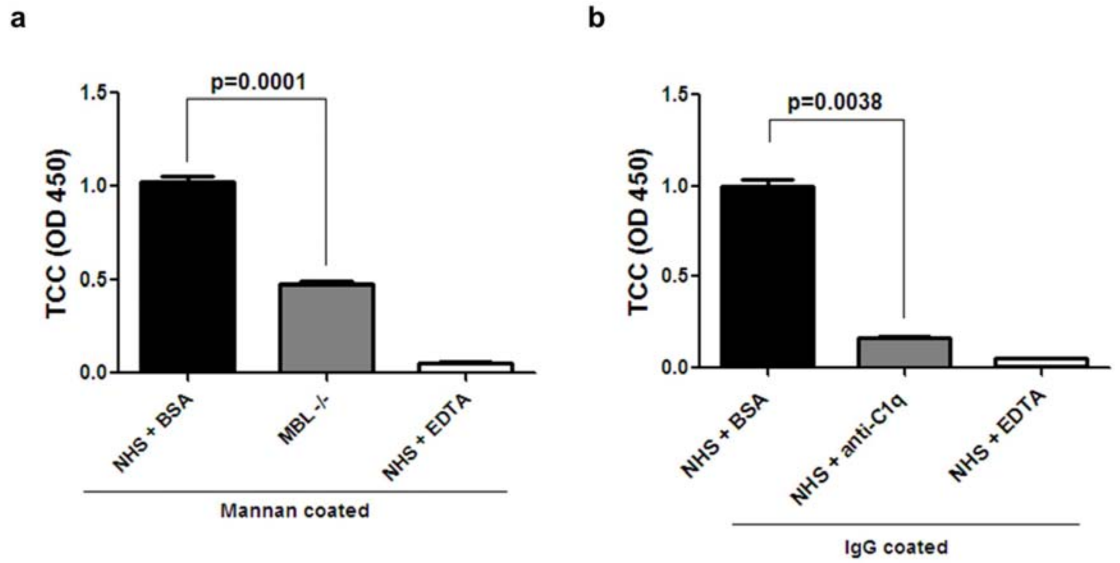

**Fig S2.**

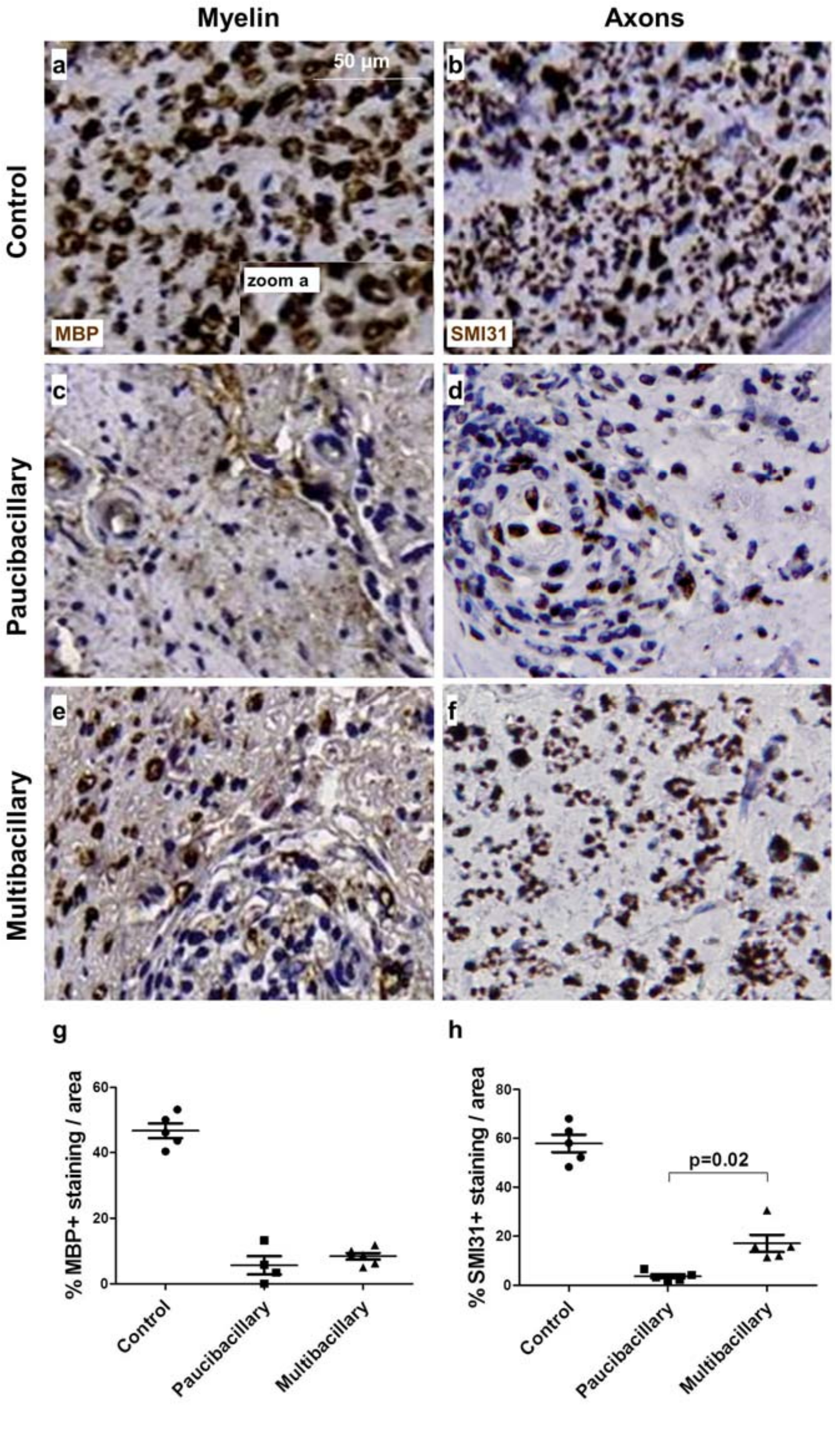
Myelin
Axons
Control
Paucibacillary
Multibacillary
a
50 µm
zoom a
MBP
b
SMI31
c
d
e
f
g
h
% MBP+ staining / area
60
40
20
0
Control
Paucibacillary
Multibacillary
% SMI31+ staining / area
80
60
40
20
0
p=0.02
Control
Paucibacillary
Multibacillary

**Fig S3.**

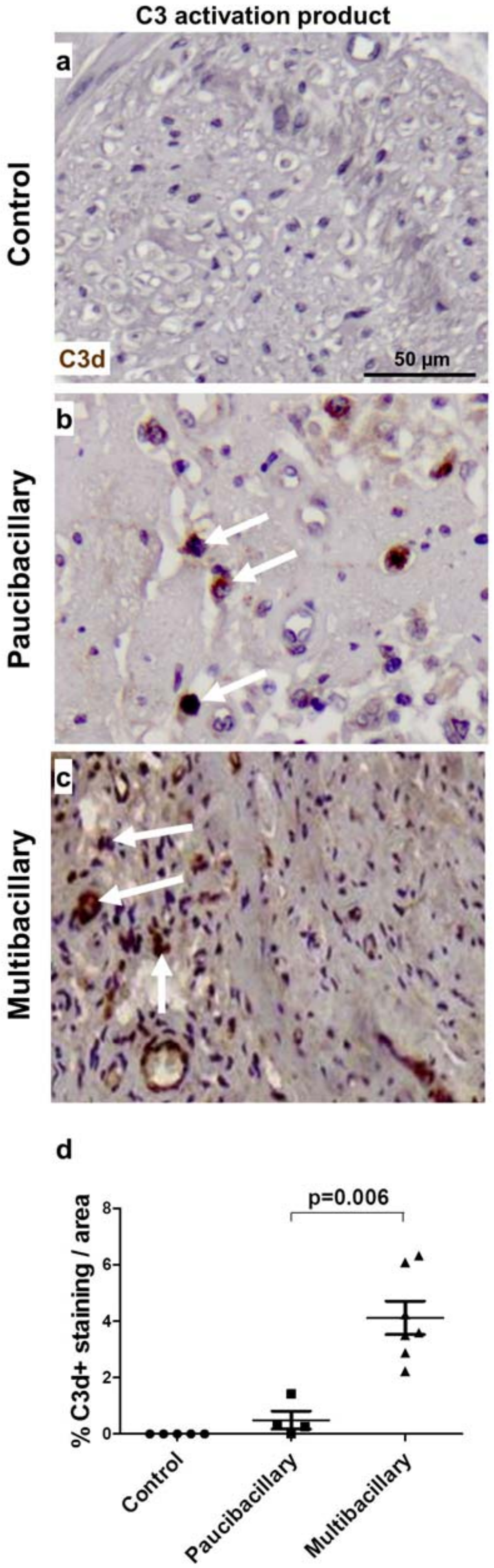

C3 activation product
a
Control
C3d
50 µm
b
Paucibacillary
c
Multibacillary
d
p=0.006
% C3d+ staining / area
8
6
4
2
0
Control
Paucibacillary
Multibacillary

**Fig S4.**

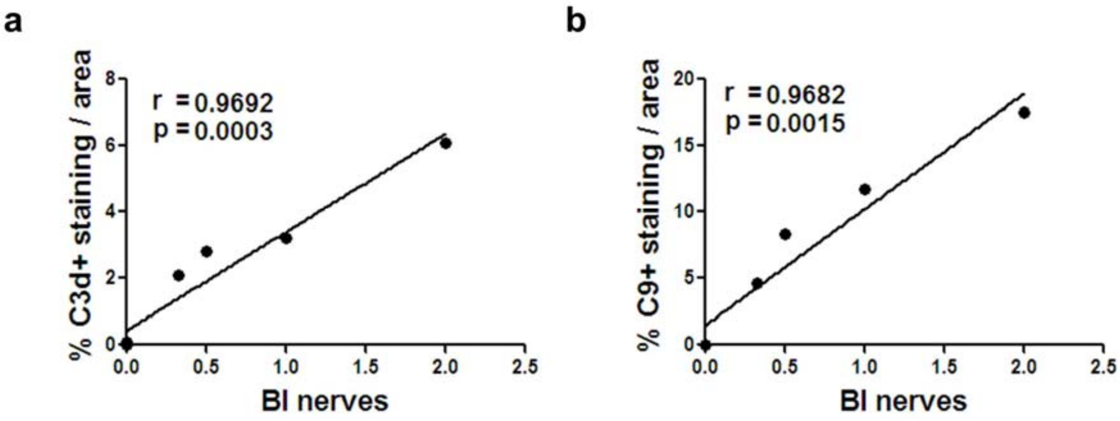
a
r = 0.9692
p = 0.0003
% C3d+ staining / area
BI nerves
b
r = 0.9682
p = 0.0015
% C9+ staining / area
BI nerves
