# Supplementary material for: M. leprae components induce nerve damage by complement activation: identification of lipoarabinomannan as the dominant complement activator
Source: Acta Neuropathol. 2015 Mar 15;129(5):653–67. doi: 10.1007/s00401-015-1404-5 (PMC4405335; doi:10.1007/s00401-015-1404-5)
Supplement: Supplementary file 3 — Supplementary material 3 (PDF 14 kb) [file 401_2015_1404_MOESM3_ESM.pdf]

***M. leprae* components induce nerve damage by complement activation: Identification of lipoarabinomanan as the dominant complement activator**, Acta Neuropathologica, Nawal Bahia El Idrissi, Pranab K. Das, Kees Fluiter, Patricia S. Rosa, Jeroen Vreijling, Dirk Troost, B. Paul Morgan, Frank Baas and Valeria Ramaglia; Corresponding author Prof. Frank Baas, email: f.baas@amc.nl.

Fig S1. a ELISA for MAC generation on mannan (1 µg)-coated plates incubated with either normal human serum (NHS) or MBL-deficient (MBL-/-) serum for 15 minutes at 37°C, showing significant reduction of MAC formation in MBL-/- serum, demonstrating that MBL-/- serum blocks lectin pathway activation initiated by mannan. **b** ELISA for MAC generation on $IgG_{1, 2, 3, 4}$ (IgG, 1 µg)-coated plates incubated with NHS with either the neutralizing anti-C1q antibody or BSA as control, showing a significant reduction of MAC formation by the anti-C1q antibody, demonstrating the anti-C1q antibody blocks classical complement activation initiated by IgG. Normal human serum with BSA produced abundant MAC generation (positive control) whereas normal human serum with EDTA (negative control) blocked MAC formation as expected.

Fig S2. Myelin loss and axonal damage in nerves of leprosy patients. Immunohistochemistry for MBP and the phosphorylated-neurofilament marker of axons, SMI31, showing intact myelin and axons in control nerves (**zoom a, b**) whereas myelin loss and axonal damage are detected in nerves of paucibacillary (**c, d**) and mutibacillary (**e, f**) leprosy patients. Quantification of the immunostainings shows reduced immunoreactivity for MBP (**g**) and SMI31 (**h**) in both paucibacillary and multibacillary nerves, indicating myelin loss and axonal damage in both groups. Notably, the amount of axonal antigens are significantly more abundant in the multibacillary nerves compare to the paucibacillary (**h**) (Student's t-test paucibacillary versus multibacillary: p=0.02). Error bars indicate standard error of the mean.

Fig. S3. C3d deposition in nerves of leprosy patients. Immunohistochemistry for C3d on nerve biopsies of controls (**a**) compared to paucibacillary (**b**) and multibacillary (**c**) leprosy patients. The control nerves are negative for C3d (**a**) whereas the nerves of paucibacillary and multibacillary

patients show immunoreactivity for C3d (**b, c and arrows**). Quantification of the staining (**d**), shows a significant higher amounts of C3d deposits in the nerves of multibacillary compared to paucibacillary patients (Student's t-test paucibacillary versus multibacillary: p=0.006). Error bars indicate standard error of the mean.

Fig. S4. Bacterial Index (BI) is associated with C3d and MAC deposition in nerves of leprosy patients. The amount of C3d (**a**) and C9 (**b**) immunoreactivity significantly correlated with the BI of paucibacillary and multibacillary leprosy nerves (Pearson's correlation, r=0.9692, p=0.0003 and r=0.9682, p=0.0015 respectively), indicating an association between the *M.leprae* BI and complement activation in leprosy nerves.
